# Development of hepatocellular adenomas in a patient with glycogen storage disease Ia treated with growth hormone therapy

**DOI:** 10.1002/jmd2.12381

**Published:** 2023-08-18

**Authors:** David G. Jackson, Rebecca L. Koch, Surekha Pendyal, Robert Benjamin, Priya S. Kishnani

**Affiliations:** ^1^ Division of Medical Genetics, Department of Pediatrics Duke University Medical Center Durham North Carolina USA; ^2^ Department of Endocrinology Duke University Medical Center Durham North Carolina USA

**Keywords:** glycogen storage disease, glycogen storage disease Ia, growth hormone therapy, hepatocellular adenoma, von Gierke disease

## Abstract

Glycogen storage disease Ia (GSD Ia), also known as von Gierke disease, is caused by pathogenic variants in the *G6PC1* gene (OMIM 232200) which encodes glucose‐6‐phosphatase. Deficiency of glucose‐6‐phosphatase impairs the processes of gluconeogenesis and glycogenolysis by preventing conversion of glucose‐6‐phosphate to glucose. Clinical features include fasting hypoglycemia, lactic acidosis, hypertriglyceridemia, hyperuricemia, hepatomegaly, and development of hepatocellular adenomas (HCAs) with potential for malignant transformation. Additionally, patients with GSD Ia often exhibit short stature, in some instances due to growth hormone (GH) deficiency. Patients with short stature caused by GH deficiency typically receive GH injections. Here, we review the literature and describe a female with GSD Ia who had short stature, failure of growth progression, and suspected GH deficiency. This patient received GH injections from ages 11 to 14 years under careful monitoring of an endocrinologist and developed HCAs during that time. To date, there is no reported long‐term follow up data on patients with GSD Ia who have received GH therapy, and therefore the clinical outcomes post‐GH therapy are unclear.

**Synopsis**

A female patient with glycogen storage disease type Ia and growth hormone deficiency was treated with growth hormone therapy in adolescence and developed hepatocellular adenomas.

## 1 INTRODUCTION

Glycogen storage disease Ia (GSD Ia, also known as von Gierke disease, OMIM #232200) is an autosomal recessive inborn error in metabolism caused by pathogenic variants in *G6PC1* which encodes the enzyme glucose‐6‐phosphatase.^1^ Deficiency of glucose‐6‐phosphatase results in a block of glycogenolysis and gluconeogenesis, leading to accumulation of glycogen and lipids in hepatocytes, intestinal epithelial cells, and proximal renal tubule cells.^2^, ^3^ The clinical features of GSD Ia include hypoglycemia and hepatomegaly which are typically first noticed at age 3–6 months of age; additional features include lactic acidemia, hypertriglyceridemia, hypercholesterolemia, hyperuricemia, osteopenia, and polycystic ovarian syndrome.^1^, ^4^ Within the liver, another significant concern remains: development of hepatocellular adenomas (HCAs).

HCAs in patients with GSD Ia typically manifest between puberty and early adulthood, though there are patients who have presented earlier in life,^1^, ^5^, ^6^, ^7^, ^8^ and they are notably different from HCAs found in the general population, including a lack of predilection for female patients, bilobar distribution, and a greater total number of HCAs as opposed to the encapsulated single HCA that result from oral contraceptive pills (OCP).^1^ Additionally, HCA associated with GSD Ia can have an atypical or “bullseye” appearance on MRI as opposed to the “atoll” sign observed in HCA in the general population.^9^, ^10^ Long‐term complications of HCA in GSD Ia include an increased risk for bleeding and potential for malignant transformation into hepatocellular carcinoma (HCC),^11^ and can lead to patients undergoing resection of large HCA or orthotopic liver transplantation if tumor burden is significant enough.

Individuals with GSD I frequently have short stature with an estimated incidence of 46.3% compared to 2.5% of adults overall.^12^ Though growth can improve with treatment, affected individuals are typically more than one standard deviation below the mean population height.^13^ The most common causes of short stature in GSD Ia are constitutional growth delay (CGD) and nutritional insufficiency. With CGD, weight and growth velocities are normal, and catch‐up growth and puberty happen later in adolescence. Nutritional insufficiency may occur with poor metabolic control in GSD, affecting weight gain and limiting substrate for normal growth.^1^

Growth hormone (GH) deficiency, while less common, can cause short stature and slowed growth velocity in patients with GSD Ia. GH is synthesized in the pituitary gland and is released in pulsatile fashion. GH stimulates the production of insulin‐like growth factor 1 (IGF‐1) and its principal serum binding protein IGFBP‐3. GH deficiency can be diagnosed by low IGF‐1 and IGFBP‐3 levels as well as by reduced GH levels with provocative testing in the context of delayed pubertal status.^14^ Patients with short stature, poor growth velocity, and suspected GH deficiency based on laboratory investigations can be placed on supplemental GH therapy with the goal of ensuring proper growth.

Administration of exogenous GH to patients with GSD Ia may increase risk of HCA formation through various mechanisms additive to the tumorigenic risks inherent in GSD Ia.^1^, ^15^ Here, we describe a female patient with GSD Ia and GH deficiency (low IGF‐1 and low GH on provocative testing) who was treated with supplemental GH injections and subsequently developed HCAs. We review the literature to compare our findings to other known hepatic GSD patients who were treated with GH therapy.

## 2 CASE REPORT

A female infant of Ashkenazi Jewish descent exhibited persistent emesis and failure to thrive at 3.5 weeks of life. A liver biopsy at 5 months old revealed “features consistent with GSD Ia.” Genetic testing identified homozygous c.247C>T (p.R83C) pathogenic variants in *G6PC1*, confirming the GSD Ia diagnosis. The patient was first evaluated by Duke University Metabolic Genetics at age 3 years, at which point she was already consuming supplemental uncooked cornstarch (UCCS). Anthropometric measurements, laboratory investigations (total cholesterol, triglycerides, lactate, uric acid, IGF‐1), diet management information, and results from semi‐annual abdominal imaging studies were available beginning at age 3 years (Tables 1 and 2).

**TABLE 1:** Anthropometric measurements and laboratory investigations from before, during, and after growth hormone (GH) therapy.

Age (years)	Height (cm)^a^	Total cholesterol (mg/dL)^b^	Triglycerides (mg/dL)^c^	Lactate (mmol/L)^d^	Uric acid (mg/dL)^e^	IGF‐1 (ng/mL)^f^	Diet management
4	90 [<5th]	283	892	N/A	N/A	N/A	UCCS^g^
5	101.6 [10th]	181	237	2.4	5.6	N/A	
7	109 [<5th]	249	799	N/A	6.8	N/A	
8	N/A	211	280	2.1	N/A	N/A	UCCS 8×day, including overnight
9.3	117 [<5th]	N/A	584	N/A	5.4	N/A	N/A
10.9	126 [<5th]	268	805	N/A	N/A	61	UCCS 3×/day, overnight tube feeding of Vivonex
**11.2** ^h^	**GH therapy initiated**						
12.0^i^	131.7 [<5th]	308	817	N/A	7.5	N/A	N/A
12.4	136.6 [<5th]	N/A	N/A	N/A	N/A	351	N/A
12.8	138.6 [<5th]	N/A	N/A	N/A	N/A	N/A	N/A
13	140.8 [<5th]	255	1151	4.3	9.8	249	UCCS 3×/day, overnight tube feeding of Vivonex
13.2	143.3 [<5th]	340	1508	N/A	N/A	512	N/A
14.3	144.6 [<5th]	303	1201	8.9	11.0	431	N/A
**14.8**	**GH therapy discontinued**						
15.2	147.0 [<5th]	N/A	N/A	5.4	7.5	N/A	UCCS every 3 hours during the day, overnight tube feeding of Vivonex
**16**	**HCA resection performed**						
19	N/A	N/A	N/A	N/A	N/A	N/A	UCCS 3–4×/week at bedtime, not taking daytime UCCS
20.5	146.7 [<5th]	230	444	8.1	8.5	N/A	Started early morning UCCS, reinitiated overnight tube feeding of Vivonex
21.5	146.5 [<5th]	295	881	2.1	7.4	N/A	
22.5	147.2 [<5th]	271	1041	5.2	7.5	N/A	Early morning UCCS, overnight tube feeding of Tolerex
23.5	147.0 [<5th]	N/A	N/A	6.1	8.7	N/A	Discontinued overnight tube feeding, reinitiated taking UCCS at bedtime and then early morning; not taking daytime UCCS
24.5	146.8 [<5th]	287	853	6.1	7.2	N/A	
25.5	146.7 [<5th]	286	859	6.5	8.2	N/A	
**26.3**	**HCA embolization performed**						
26.7^j^	147.3 [<5th]	208	519	3.4	9.8	N/A	N/A
**26.7**	**Orthotopic liver transplantation performed**						
27.7	148.6 [<5th]	124	155	4.8	5.4	N/A	Normal diet
28.5	148.6 [<5th]	187	124	0.7	9.0	N/A	
30.5	148.6 [<5th]	174	101	0.7	N/A	N/A	

*Note*: Findings are described before initiation of, during administration of, and after discontinuation of GH therapy. All laboratory investigations were performed at routine clinic visits when the patient was considered stable. Brackets indicate age‐matched percentiles for height. N/A indicates test was not performed/results were not available (anthropometrics and laboratory investigations) or information was not available (diet management). Bold text defines ages at which GH therapy was initiated and discontinued, when hepatocellular adenomas (HCAs) were resected and embolized, and when liver transplantation was performed.

Abbreviations: IGF‐1, insulin‐like growth factor 1; UCCS, uncooked cornstarch.

^a^ Target (mid‐parental) height for patient was 159.77 cm or 40th percentile for adult women.

^b^ Total cholesterol reference range: desirable, <200 mg/dL; elevated: >240 mg/dL.

^c^ Triglyceride reference range: desirable, <200 mg/dL; borderline, 200–400 mg/dL; elevated, >400 mg/dL; severely elevated, >1000 mg/dL.

^d^ Lactate reference range by visit age: 5 years, 1.0–1.8 mmol/L; 8 years, no reference range documented; 13–15.2 years, 0.5–2.2 mmol/L; 20.5–30.5 years, 0.6–2.5 mmol/L.

^e^ Uric acid reference range by visit age: 5–12 years, 2–7 mg/dL; 20.5–22.5 years, 2.5–8.0 mg/dL; 23.5–28.5 years, 2.6–8.0 mg/dL.

^f^ IGF‐1 reference range: 17–485 ng/mL.

^g^ Specific UCCS dosage details unavailable.

^h^ GH therapy initiated at age 11.2 years at 0.24 mg/kg per week.

^i^ GH therapy dosage.

^j^ This set of laboratory investigations was done 7–8 days prior to orthotopic liver transplantation.

**TABLE 2:** Liver imaging studies before, during, and after growth hormone (GH) therapy.

Age (years)	Liver Imaging
3.7	U/S: marked hepatomegaly, no focal masses
5	U/S: stable hepatomegaly, no HCA noted
7.5	U/S: hepatomegaly, no concern for HCA
9	U/S: marked hepatomegaly, no focal lesions
10.9	U/S: hepatomegaly, no focal lesions CT: hepatomegaly, no evidence of HCA
**11.2** ^a^	**GH therapy initiated**
12.0^b^	N/A
13.2	CT: hepatomegaly (3050 cc total volume, 3× upper limit of normal). Small (5 mm) low‐attenuation lesion in the right lobe of the liver was not previously present and likely represents a small HCA. Additional 2–3 other tiny indeterminate lesions that were low in attenuation and a couple mm.
14.3	CT: marked hepatomegaly (total liver span 24 cm craniocaudal), several well‐defined foci of low‐density scattered throughout the liver measuring <1 cm have slightly increased in size from the prior year. A new 5 cm mass with partially defined margins and heterogenous central enhancement versus hemorrhage is seen in the peripheral inferior aspect of the left hepatic lobe lateral segment. A second new smaller 1 cm similar lesion is seen in the right hepatic lobe posterior segment.
**14.8**	**GH therapy discontinued**
**16**	**HCA resection performed**
17.5	CT: focal hepatic (postoperative) cyst and no other notable lesions
20.2	CT: hepatomegaly, stable subcentimeter low attenuation lesions in the caudate lobe and medial aspect of the right hepatic lobe
21.5	MRI: hepatomegaly diminished compared to prior study, multiple liver lesions
22.1	MRI: hepatomegaly, multiple lesions
23.6	MRI: hepatomegaly, 4 small lesions
24.3	MRI: hepatomegaly, multiple lesions
25.2	MRI: moderate to severe hepatomegaly with multiple enhancing lesions (largest 2.8 × 2.4 cm), one focus concerning for hemorrhage
26.1	MRI: marked hepatomegaly, diffuse hepatic steatosis, multiple lesions scattered throughout the liver (largest 3.8 × 4.8 cm). The largest lesion was enlarging compared to previous findings and was concerning for hemorrhage.
**26.3**	**HCA embolization performed**
26.7^c^	MRI: hepatomegaly, marked diffuse hepatic steatosis, numerous arterial enhancing lesions identified that were stable to slightly larger than on prior examination and compatible with adenomas. One lesion has significantly increased in size compared to prior exam and was worrisome for HCC.
**26.7**	**Orthotopic liver transplantation performed**

*Note*: Radiology reports of the liver, including observations of hepatocellular adenoma (HCA) or hepatocellular carcinoma (HCC), from abdominal ultrasounds (U/S), computed tomography (CT) scans with contrast, and magnetic resonance imaging (MRI) with contrast are detailed before initiation of, during, and after discontinuation of GH therapy until age at liver transplantation. N/A indicates test was not performed or results were not available. Bold text defines ages at which GH therapy was initiated and discontinued, when HCAs were resected and embolized, and when liver transplantation was performed.

^a^ GH therapy initiated at age 11.2 years at 0.24 mg/kg per week.

^b^ GH therapy dosage increased at age 12.0 years to 0.3 mg/kg per week.

^c^ MRI was performed 7–8 days prior to orthotopic liver transplantation.

The patient's clinical course was complicated by acute gastroenteritis requiring hospitalization at age 4 years. Between ages 4 and 7 years, the patient was noted to have brief, intermittent hypoglycemic episodes (approximately once per week) diagnosed based on symptoms (pallor, shaking, irritability) that would resolve with administration of UCCS. At age 8 years, she sustained a spiral fracture of her right tibia after a fall while ice skating for which she was casted for 15 weeks. She also had a metatarsal stress fracture around age 10 years. Retrospectively, these fractures were concerning for low bone mineral density.

**FIGURE 1:**
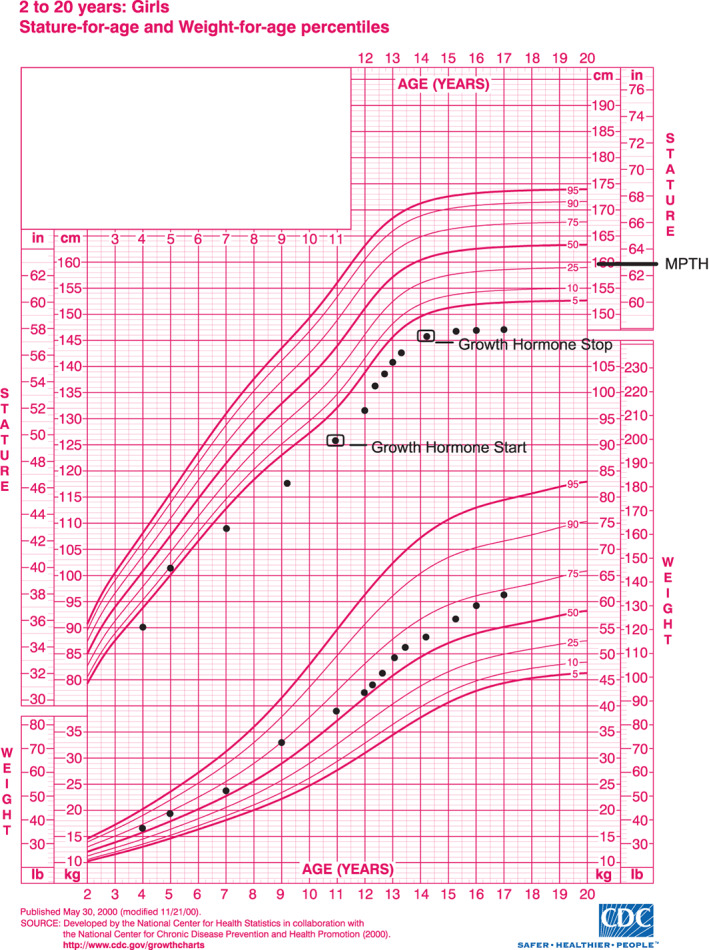
Growth chart for a patient with glycogen storage disease Ia that was treated with growth hormone therapy. See Table 1 for specific height data. Black box around height points indicate when growth hormone therapy was started and stopped, respectively. Mid‐parental target height (MPTH) is noted at 159.77 cm (63 in.). Growth chart for 2–20 years: Girls adapted from the Centers for Disease Control and Prevention.

The patient was formally evaluated by Duke University Pediatric Endocrinology at 10.9 years due to growth slowing; her growth velocity had slowed to approximately 1 inch per year and her height positioning was below her expected mid‐parental target height (MPTH) percentile (Figure 1). Her weight velocity and positioning were normal and she was continuing to supplement with UCCS and tube feeding under the direction of a metabolic dietitian. During her visit, she had breast budding (consistent with early pubertal development), low IGF‐1 levels (61 ng/mL, reference range 70–305 ng/mL), and normal thyroid studies: TSH 1.8 μIU/mL (normal 0.5–5.0 μIU/mL) and T4 1.04 ng/dL (normal 1.0–2.1 ng/dL) (Table 1). IGFBP‐3 levels were not obtained. Provocative testing of GH involved one agent (arginine at 0.05 mg/kg delivered intravenously) and resulted in peak GH level of 1.3 ng/mL, well below the cutoff of 10 ng/mL. Because the patient was in central puberty, she did not receive exogenous sex‐steroid priming with estradiol prior to the stimulation test. Due to suspected GH deficiency, the patient was started on daily recombinant injectable GH (Somatotropin, Nutropin). Initial GH dosing at age 11 years was 0.24 mg/kg per week which was increased to 0.3 mg/kg per week at age 12 years. The patient exhibited improved linear growth on this therapy but remained below the 5th percentile in height (Figure 1). By age 13 years, the patient progressed to Tanner Stage 3 breast development and growth velocity was approximately 12 cm/year (peak pubertal rate). At that time, the patient had been receiving GH therapy for approximately 2 years. Her thyroid profile remained normal and IGF‐1 levels increased appropriately from a baseline of 61 ng/mL to 249‐512 ng/mL (Table 1).

The patient continued to manifest hypertriglyceridemia and hypercholesterolemia attributed to GSD Ia with no improvement while on GH therapy. Serial abdominal ultrasound (U/S) studies from 4 to 10 years of age revealed hepatomegaly but otherwise were negative for evidence of HCA, with her last study 3 months prior to starting GH treatment. HCAs were first observed at age 13 years on a routine abdominal computed tomography (CT) scan with contrast enhancement which revealed multiple low‐density foci in the liver (Table 2). At age 14 years, abdominal CT scan revealed marked hepatomegaly and showed multiple well‐defined low‐density foci within the liver (likely HCAs) which were concerning for increased size as compared to the previous year; the largest lesion was a 5 cm mass with heterogeneity concerning for HCA with hemorrhage in the left hepatic lobe lateral segment. That CT scan also identified a new 1 cm lesion (likely HCA) in the right hepatic lobe posterior segment. Due to the notable increase in HCA size, GH therapy was discontinued. By that time, the patient had been treated with exogenous GH therapy for approximately 3.5 years, with the last 1.5 years of GH treatment occurring when she was known to have HCAs. At no point in time during GH therapy was this patient taking OCP. After discontinuing GH therapy, the patient continued to gain height until age 15 years, ultimately attaining adult height at 148.6 cm (Figure 1).

At age 16 years, the patient underwent resection of the largest HCA located in the left hepatic lobe lateral segment due to its risk for bleeding. At age 17 years, no large (>1 cm) HCAs were found on abdominal CT, but small subcentimeter lesions suspected to be HCAs were found on an abdominal CT scan done at age 20 years. Subsequent surveillance screening done through age 24 years showed stable HCAs without bleeding. Abdominal MRI performed at age 25 years revealed multiple HCAs with one possible area of hemorrhage, and at age 26 years, an abdominal MRI revealed multiple enlarging HCAs with concern for hemorrhage. Shortly after detection, the largest HCA was treated with embolization therapy. Despite embolization, the patient had spontaneous rupture and bleeding from an HCA within 2–3 weeks of the embolization procedure. Approximately 5 months after the embolization procedure, the patient received an orthotopic liver transplant due to numerous scattered HCAs with concern for hemorrhage and malignant transformation to HCC.

Immediately prior to transplantation, the patient was notable for hypertriglyceridemia (519 mg/dL) and elevated uric acid (9.8 mg/dL), indicating poor metabolic control. Explant pathology indicated that the lesions were non‐encapsulated, homogenous and without malignant transformation. At age 31 years, the patient gave birth to a healthy child. She is now 36 years old, following an immunosuppression regimen of tacrolimus as indicated for post‐liver transplantation.

## 3 DISCUSSION

GSD Ia is an inherited metabolic disorder causing deficient levels of glucose‐6‐phosphatase, impairing glycogenolysis and gluconeogenesis and resulting in numerous metabolic derangements including life threatening fasting hypoglycemia, lactic acidosis, hyperuricemia, and hypertriglyceridemia.^1^ Systemic features associated with GSD Ia include hepatomegaly, short stature, and formation of HCA. While many HCAs are benign and amenable to surgical resection, care should be directed toward minimizing HCA formation, as these lesions confer risk with an increase in size and number of lesions, risk for significant bleeding and hemorrhage, and malignant transformation into HCC.^16^

GH deficiency has been described in multiple forms of hepatic GSD, including types Ia, Ib (glucose‐6‐phosphate transporter deficiency), III (glycogen debranching enzyme deficiency), and IX (phosphorylase kinase deficiency) (Table 3). It remains unknown how interference with the various steps of glycogen mobilization contributes to GH deficiency. Once the diagnosis of GH deficiency is made, risks and benefits of GH therapy should be considered. Untreated GH deficiency may lead to symptomatic hypoglycemia, growth failure, and prohibitive short stature in adulthood. Our patient received GH injections due to short stature, growth failure with normal weight positioning, low IGF‐1 levels, and failed GH stimulation testing. While receiving standard GH therapy, the patient exhibited growth acceleration but developed HCAs after 2 years of injections.

**TABLE 3:** Review of the literature of patients with a hepatic glycogen storage disease (GSD) who received growth hormone (GH) therapy.

GSD type	*N*	Sex	Therapy initiation	Therapy duration	Last follow‐up	HCA formation	Reference
I^a^	1	M	16	“Several years”	23	Not mentioned	Bierich^21^
	2	M	Not stated	“Several years”	Not stated	Not mentioned	
Ia	1	M	10.9	1.1	12	No HCA	Wu et al.^22^
Ib	1	M	9.8	3.0	12.8	Not mentioned	Zhang et al.^23^
Ib	1	F	13	2.2	17	Not mentioned	Noto et al.^24^
III	1	M	16.6	0.17^b^	17.3	Not mentioned	Larizza et al.^25^
IX α2	1	M	3.9	Not mentioned	3.9	Not mentioned	Hodax et al.^26^

*Note*: A search of the literature was undertaken to identify all published cases with a hepatic GSD that received GH therapy. Using a strategy designed by a Duke University School of Medicine medical librarian, the MEDLINE database via Ovid was searched for relevant publications, as detailed in Appendix S1. References identified by this search were further reviewed and duplicates were removed. The remaining papers were evaluated by two independent reviewers to assess their eligibility for inclusion. Papers were excluded if the full text could not be located in English or if no clinical data were reported. “Hepatic GSD” was defined as GSD types 0a, Ia, Ib, III, IV, VI, and IX. Patients diagnosed based solely on histological or physical findings (i.e., without genetic or enzymatic confirmation) were excluded. Age at GH therapy initiation, duration of GH therapy, and age at last reported follow‐up visit are provided in years. Formation of hepatocellular adenoma (HCA) is noted. *N* indicates number of reported patients.

^a^ Type of GSD I (Ia or Ib) was not specified.

^b^ GH therapy was discontinued due to extreme hyperlipidemia.

There is a paucity of long‐term data investigating GH therapy and HCA formation in hepatic GSD patients. Overall, there are very few studies investigating the impact of GH therapy in patients with hepatic GSD and GH deficiency (Table 3); lack of notable HCA formation in prior studies may be due to the limited reported monitoring period. To date, there has been no reported systemic prospective study evaluating the occurrence of HCA formation in patients with a hepatic GSD who have received GH therapy. Therefore, at this time, there no clear relationship between GH therapy and HCA development in hepatic GSD.

A proposed mechanism for HCA formation in GSD I patients on GH therapy includes increased expression of galectin genes which are implicated in tumor cell migration.^17^, ^18^ A mouse model over‐expressing GH had markedly elevated galectin levels^17^ and advanced‐stage HCC is often characterized by increased galectin expression.^17^, ^19^ It is also possible that GH therapy in hepatic GSD leads to altered glucose metabolism and may confer different risk depending on whether gluconeogenesis, glycogenolysis, or both processes are impaired.

While the timing of our patient's tumors implied a contribution from GH therapy, there were limitations in her workup of GH deficiency and there were several other risk factors for HCA development. The patient did not have IGFBP‐3 levels drawn which are less sensitive to metabolic changes than IGF‐1. Additionally, she did not have a second provocative agent added to her GH stimulation testing and she did not have a pituitary MRI prior to starting GH therapy. The patient had gradually worsening metabolic control which is a major risk factor for HCA. Moreover, the patient was in central puberty, a time of increased risk for HCA formation in GSD I^1^ potentially from sex steroid contribution to HCA independent of GH therapy. Lastly, we cannot rule out if the patient had small HCA prior to starting GH therapy, as small hepatic lesions are difficult to detect on routine U/S. Additional data is needed to better understand the contribution, and potentially additive effects, of poor metabolic control, sex steroids, and GH therapy on formation and progression of HCA in hepatic GSDs.

Additional studies investigating the frequency of HCA by genotype are warranted. Our patient was homozygous for the *G6PC* c.247C>T (p.R83C) pathogenic variant which is highly prevalent^20^ and associated with HCA development in puberty.^8^ Prospective studies, particularly systematic studies including patients from multiple centers, should also be performed for patients with a hepatic GSD that are receiving GH therapy in order to delineate the natural history of HCA formation in these subsets of patients.

Guidelines should be established for the treatment of hepatic GSD patients with confirmed GH deficiency to enable achievement of growth potential without exacerbating the risk of HCA formation. Careful monitoring with systematic collection of biomarkers, assessment of metabolic control, and involvement of endocrinology in decision‐making is essential for the treatment of GH deficiency to ensure proper metabolic control and mitigate the risk of HCA formation. In closing, our case stresses the importance of comprehensive, long‐term monitoring of all patients with GSD Ia who are receiving GH therapy.

## AUTHOR CONTRIBUTIONS

Supervision: Priya S. Kishnani; Writing original draft: David G. Jackson, Rebecca L. Koch; Literature review: David G. Jackson, Rebecca L. Koch; Writing, review, and editing: David G. Jackson, Rebecca L. Koch, Surekha Pendyal, Robert Benjamin, Priya S. Kishnani.

## FUNDING INFORMATION

The authors declare no funding sources.

## CONFLICT OF INTEREST STATEMENT

The authors declare that the research was conducted in the absence of any commercial or financial relationships that could be construed as a potential conflict of interest.

## ETHICS

This case report was conducted in accordance with the Duke University Health System (DUHS) Institutional Review Board (IRB). The review of medical records for publication of a single case report is not considered by the DUHS IRB to be research involving human subjects, and therefore this medical case report did not require IRB review and approval. The collection and evaluation of all protected patient health information was performed in a Health Insurance Portability and Accountability Act (HIPAA)‐compliant manner. All procedures followed were in accordance with the ethical standards of the responsible committee on human experimentation (institutional and national) and with the Helsinki Declaration of 1975, as revised in 2000 (5).

## Supporting information


**Appendix S1:** Supporting Information.

## Data Availability

The original contributions presented in the case report are included in the article. Further inquiries can be directed to the corresponding author.
