# Supplementary material for: Development of hepatocellular adenomas in a patient with glycogen storage disease Ia treated with growth hormone therapy
Source: JIMD Rep. 2023 Aug 18;64(5):303–11. doi: 10.1002/jmd2.12381 (PMC10494510; doi:10.1002/jmd2.12381)
Supplement: Supplementary file 1 — Appendix S1: Supporting Information. [file JMD2-64-303-s001.docx]

**Literature Review Search Strategy.** Designed by Elizabeth Blackwood, B.A. at Duke University.

Database: MEDLINE via Ovid

| **Concept** | **Strategy** | **Results** |
| --- | --- | --- |
| #1 *Glycogen Storage Disease* | "Glycogen Storage Disease"[Mesh] OR "Glycogen Storage Disease"[tiab] | 7,331 |
| #2 *Growth Hormone Therapy* | "Growth Hormone"[Mesh] OR "growth hormone"[tiab] | 76,429 |
| #3 *Combined* | #1 AND #2 | 49 |
| Validation String | 9290603 OR 27041087 OR 12948077 OR 20022338 OR 31617422 | 5/5 |
